# SOX combined with sintilimab versus SOX alone in the perioperative management of locally advanced gastric cancer: a propensity score–matched analysis

**DOI:** 10.1007/s10120-023-01431-z

**Published:** 2023-09-28

**Authors:** Xingmao Huang, Jingquan Fang, Ling Huang, Hang Chen, Han Chen, Tengjiao Chai, Zeyao Ye, Hanguang Chen, Qi Xu, Yian Du, Pengfei Yu

**Affiliations:** 1 Department of Gastric Surgery, Zhejiang Cancer Hospital, Hangzhou Institute of Medicine (HIM), Chinese Academy of Sciences, Hangzhou, 310022 Zhejiang China; 2 Postgraduate Training Base Alliance of Wenzhou Medical University (Zhejiang Cancer Hospital), Hangzhou, 310022 Zhejiang China; 3 Zhejiang Chinese Medical University, Hangzhou, 310053 Zhejiang China; 4 Department of General Surgery, No. 2 People’s Hospital of Yuhang District, Hangzhou, 310022 Zhejiang China; 5 Department of Medical Oncology, Zhejiang Cancer Hospital, Hangzhou Institute of Medicine (HIM), Chinese Academy of Sciences, Hangzhou, 310022 Zhejiang China

**Keywords:** Chemotherapy, Gastric cancer, Immune checkpoint inhibitor, Pathological response, Prognosis

## Abstract

**Objectives:**

To evaluate the efficacy of SOX combined with a programmed cell death protein-1 (PD-1) inhibitor compared with SOX alone in the perioperative management of locally advanced gastric cancer and to explore biomarkers that may predict response to anti-PD-1 therapy.

**Methods:**

Data of patients with clinical stage T3–4aN0–3M0 (IIb–III) gastric cancer were reviewed to create a primary database. Patients treated with perioperative SOX combined with sintilimab were included in Group A, while those treated with SOX alone were included in Group B. After one-to-one propensity score matching, pathological response and short-term survival outcomes were compared between the two groups. In addition, potential efficacy-related biomarkers were analyzed.

**Results:**

Between January 2018 and December 2022, a total of 150 patients were included in the analysis, with 75 patients in each group. The rates of pathological complete response (21.3% vs. 4.0%; P = 0.001) and major pathological response (45.3% vs. 22.7%; P = 0.003) in Group A were statistically higher than those in Group B. There was no significant difference in 1-year overall survival (92.8% *vs*. 92.0%; *P* = 0.392) and disease-free survival (88.9% *vs*. 88.0%; *P* = 0.357) between the two groups. Subgroup analysis of Group A showed that the pathological complete response (40.6% vs. 8.6%; P = 0.002) and major pathological response (65.6% vs. 28.6%; P = 0.002) rates were significantly higher in programmed death ligand-1-positive patients with a combined positive score of ≥ 5. A pathological complete response was achieved in 42.9% patients (3/7) with mismatch repair deficiency. For the two patients confirmed as Epstein-Barr virus-positive, one patient achieved a pathological complete response and the other achieved a major pathological response.

**Conclusions:**

The adoption of SOX combined with a PD-1 inhibitor may improve the pathological response rate of patients with locally advanced gastric cancer, especially those with programmed death ligand-1 combined positive score ≥ 5, Epstein–Barr virus-positivity and mismatch repair deficiency. However, further prospective studies are still warranted to confirm the long-term survival benefit.

**Supplementary Information:**

The online version contains supplementary material available at 10.1007/s10120-023-01431-z.

## Introduction

Gastric cancer ranks as the fifth most common malignancy and the fourth most common cause of cancer death around the world [1]. Most patients are diagnosed at an advanced stage. Radical gastrectomy is the main treatment strategy for locally advanced gastric cancer (LAGC) [2, 3]. However, postoperative tumor recurrence and distant metastasis are common and can lead to poor prognosis [4, 5].

Different management strategies have been proposed to improve the prognosis of patients with LAGC, and perioperative chemotherapy has increasingly become an important treatment modality [6]. Studies have shown that preoperative chemotherapy can eliminate occult micrometastases, achieve tumor downstaging, and improve the curative resection rate [7–9]. Additional studies have shown that perioperative chemotherapy can improve the prognosis of patients with LAGC compared with adjuvant chemotherapy alone [8, 10]. However, the relatively low pathological complete response (pCR) rate was unsatisfactory, which may be caused by the high heterogeneity of gastric cancer.

The introduction of immune checkpoint inhibitors (ICIs) has provided clinical benefits for patients with gastric cancer, especially those with late-stage disease [11–13]. As ICIs, programmed cell death protein-1 (PD-1) inhibitors elicit a strong immune response by blocking the interaction of PD-1 with its ligands, programmed death ligand-1 [PD-L1] and PD-L2, which are highly expressed on cancer cells [14]. The CheckMate-649 trial reported that PD-1 inhibitors in combination with chemotherapy improved the overall survival (OS) and progression free survival (PFS) of patients with advanced gastric, gastroesophageal junction, or esophageal adenocarcinoma, especially for those with PD-L1 combined positive score (CPS) ≥ 5 [12]. Anti-PD-1 therapy also exerts a positive effect on patients with LAGC. The addition of PD-1 inhibitors to cytotoxic chemotherapy as neoadjuvant treatment for LAGC showed encouraging results in terms of pathological response [15–18]. However, most studies were single-arm studies with small sample sizes, and there is a lack of effective biomarkers for predicting the efficacy of anti-PD-1 therapy.

We conducted a retrospective study to evaluate the efficacy of SOX combined with sintilimab compared with SOX alone in the perioperative management of patients with stage IIb–III LAGC using propensity score matching (PSM) and to explore biomarkers that may predict response to anti-PD-1 therapy.

## Methods

### Study design

This was a single-center, retrospective study conducted using PSM. The study aimed to compare the short-term effects of perioperative chemotherapy plus a PD-1 inhibitor *versus* chemotherapy alone in patients with stage IIb–III gastric cancer.

The data of 897 patients with LAGC who received perioperative treatment at the Department of Gastric Surgery, Zhejiang Cancer Hospital, China, between January 2018 and December 2022 were collected and reviewed to create an analytical database. The main selection criteria were: aged 18 to 75 years, histological diagnosis of gastric cancer, clinical stage T3–4aN0–3M0 (IIb–III) disease, received perioperative treatment (SOX plus sintilimab/SOX alone) and radical gastrectomy, Eastern Cooperative Oncology Group performance status 0–1, with adequate organ function to tolerate chemoimmunotherapy or surgery. Patients were excluded if they had remnant gastric cancer, concomitant malignancies or refractory autoimmune diseases, a long-term history of steroid or immunosuppressant use, or missing clinicopathological data. A total of 658 patients were excluded due to the adoption of other treatment modalities (n = 419), loss to follow-up (n = 97), palliative-intent gastrectomy (n = 49), insufficient data (n = 81), and incomplete treatment (n=12). The remaining 239 patients who met the selection criteria were enrolled. Patients treated with perioperative SOX plus sintilimab were included in Group A (n = 79), while those treated with SOX alone were included in Group B (n = 160).

**Fig. 1:**
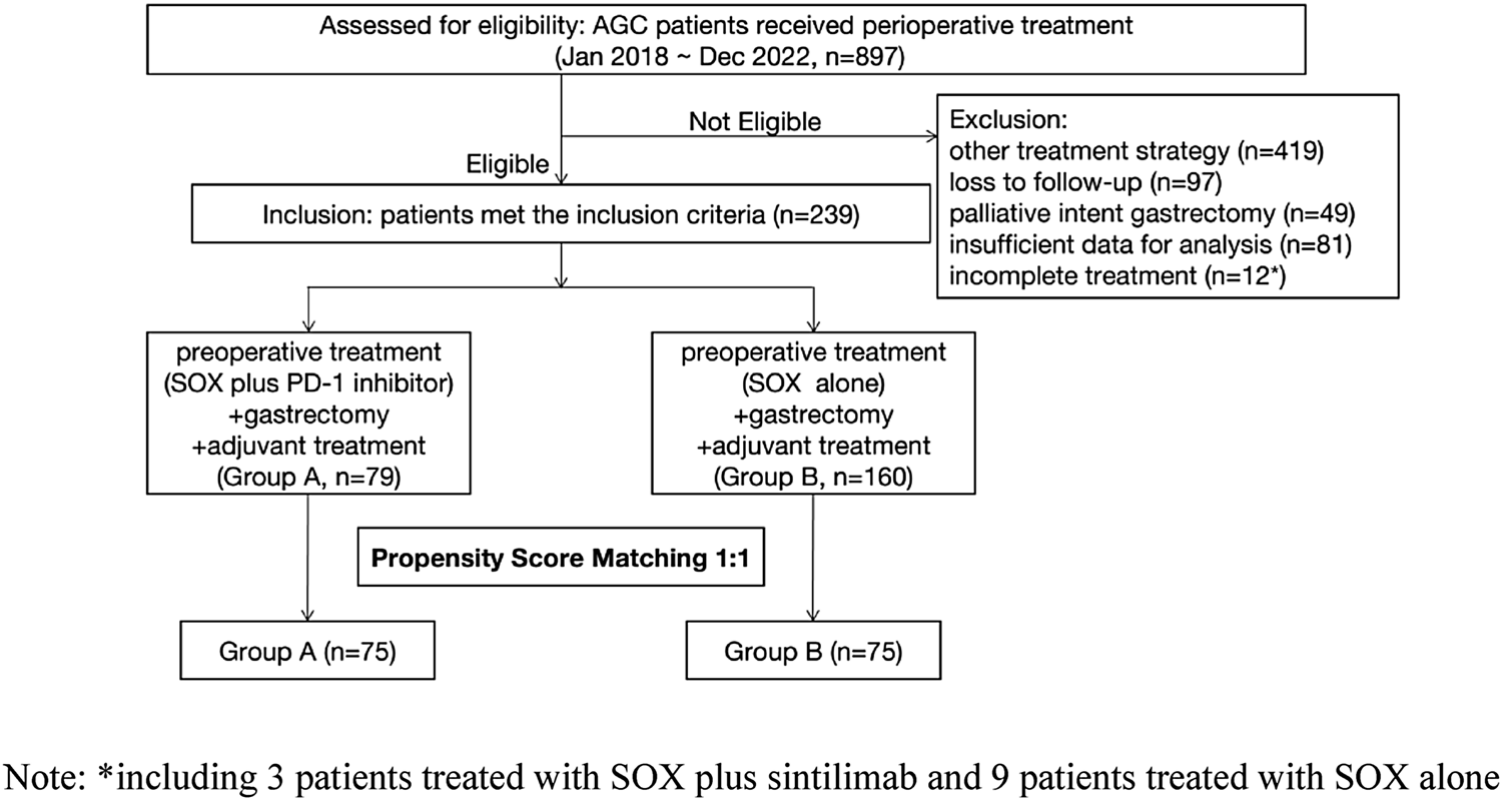
Flow diagram of the patient selection process. *Including 3 patients treated with SOX plus sintilimab and 9 patients treated with SOX alone

In order to reduce selection bias and ensure comparability between the two groups, PSM without replacement was performed in a 1:1 ratio with a 0.05 caliper width. The resulting propensity score-matched pairs were used in subsequent analyses. Covariates included age, sex, and clinical stage, which were considered to have an effect on the study endpoints. Balance was checked by computing absolute standardized mean differences (SMD) between the two groups for each covariate in the PSM sample. A SMD threshold of 0.2 was considered to detect substantial imbalance. After PSM, the propensity score-matched cohort comprised 75 patients in each group and the baseline variables between the groups were comparable (P > 0.05, SMD < 0.200, Table 1). The process of patient selection is presented in Fig. 1.

**Table 1:** Baseline characteristics before and after propensity score matching (PSM)

Variable	Before PSM				After PSM			
	Group A (n = 79)	Group B (n = 160)	P-value	SMD	Group A (n = 75)	Group B (n = 75)	P-value	SMD
Age (years)			0.088	0.235			0.866	0.029
≤ 60	33 (41.8)	49 (30.6)			29 (38.7)	28 (37.3)		
> 60	46 (58.2)	111 (69.4)			46 (61.3)	47 (62.7)		
Gender			0.790	0.037			0.707	0.062
Male	59 (74.7)	122 (76.3)			55 (73.3)	57 (76.0)		
Female	20 (25.3)	38 (23.8)			20 (26.7)	18 (24.0)		
Clinical stage (cTNM)			0.570	0.078			0.754	0.050
IIb	7 (8.9)	18 (11.3)			5 (6.7)	6 (8.0)		
III	72 (91.1)	142 (88.8)			70 (93.3)	69 (92.0)		
Preoperative CEA			0.787	0.039			0.402	0.136
≤ 5	61 (77.2)	126 (78.8)			59 (78.7)	63 (84.0)		
> 5	18 (22.8)	34 (21.3)			16 (21.3)	12 (16.0)		
Preoperative CA125			0.167	0.185			0.239	0.191
≤ 35	65 (82.3)	142 (88.8)			62 (82.7)	67 (89.3)		
> 35	14 (17.7)	18 (11.3)			13 (17.3)	8 (10.7)		
Preoperative CA199			0.371	0.123			0.577	0.091
≤ 37	56 (70.9)	122 (76.3)			54 (72.0)	57 (76.0)		
> 37	23 (29.1)	38 (23.8)			21 (28.0)	18 (24.0)		

*CEA* carcinoembryonic antigen, *CA125* carbohydrate antigen 125, *CA199* carbohydrate antigen199

This study was approved by the Ethics Committee of Zhejiang Cancer Hospital (approval number: IRB-2022-592) and was conducted in accordance with the Declaration of Helsinki. Informed consent was obtained from each patient enrolled in the study.

### Treatment

#### Perioperative  chemotherapy and immunotherapy

SOX plus sintilimab was administered to patients in Group A, while those in Group B were treated with SOX alone. Patients received 2–4 cycles of preoperative treatment and 4–6 cycles of postoperative treatment, with each cycle lasting 3 weeks. The dose of SOX regimen was calculated according to body surface area (BSA): oxaliplatin (130 mg/m^2^) was administered intravenously on Day 1 and S-1 (BSA < 1.25 m^2^, 80 mg/day; 1.25 ≤ BSA < 1.50 m^2^, 100 mg/day; BSA ≥ 1.50m^2^, 120 mg/day) was administered orally on Days 1–14, followed by a 1-week rest. Sintilimab (200 mg every 3 weeks) was administered intravenously on Day 1 after the beginning of each cycle in Group A.

#### Surgery

All patients underwent D2 radical gastrectomy according to the Japanese Gastric Cancer Treatment Guidelines [19, 20]. Distal or total gastrectomy was performed according to tumor location. Different reconstruction methods, including Roux-en-Y esophagojejunostomy, Billroth II gastrojejunostomy, and Billroth I gastroduodenostomy, were performed according to the extent of gastrectomy. Resected specimens were assessed by two experienced pathologists to determine the overall response and pathological staging after neoadjuvant treatment. The residual tumor (R) classification was used to categorize tumor status after treatment. Surgical margins with no residual tumor, microscopic residual tumor, and macroscopic residual tumor were defined as R0, R1, and R2 resection, respectively [21]. Postoperative complications were graded according to the Clavien–Dindo classification [22].

### Endpoints and assessment

The pCR rate was the primary endpoint, and the major pathological response (MPR), R0 resection, disease-free survival (DFS) and OS rates were analyzed as the secondary endpoints.

Adverse events were graded according to the latest version of the National Cancer Institute’s Common Terminology Criteria for Adverse Events (CTCAE, V.3.0) [23]. Tumor response was assessed in accordance with the Response Evaluation Criteria in Solid Tumors (RECIST) guidelines (version 1.1) [24]. The responses were categorized as follows: complete response (CR), partial response (PR), stable disease (SD), and progressive disease (PD). Tumor regression grading (TRG) was adopted to evaluate the tumor regression of resected specimens. TRG was defined as follows: TRG1a, no residual tumor cells; TRG1b, < 10% residual tumor cells; TRG2, 10–50% residual tumor cells; TRG3, > 50% residual tumor cells [25]. Resected specimens without viable tumor cells were considered as pCR (TRG1a). Less than 10% residual tumor cells after preoperative treatment were considered as MPR (TRG1a/b). Downstaging was assessed by comparing the differences between clinical and pathological staging.

PD-L1 expression, mismatch repair (MMR) status, and Epstein-Barr virus (EBV)-positivity were retrospectively assessed in formalin-fixed, paraffin-embedded biopsy samples from Group A patients. PD-L1 expression was determined using immunohistochemistry (PD-L1, Clone 22C3; Dako) and described using CPS. CPS was defined as the total number of PD-L1-positive cells, including tumor cells, lymphocytes, and macrophages, divided by the total number of living tumor cells multiplied by 100. Patients with a CPS ≥ 1 were considered PD-L1-positive. MMR proteins, including MLH1 (clone ES05; Dako), MSH2 (clone FE11; Dako), MSH6 (clone EP49; Dako), and PMS2 (clone EP51; Dako), were stained by immunohistochemistry to determine MMR status. Loss of any of the four MMR proteins was defined as MMR deficiency (dMMR). Additionally, in situ* hybridization* was performed to evaluate the EBV status (EBV-0050; EBER ISH Kit, MXB).

### Follow-up

After treatment, follow-up was performed every 3 months for the first 2 years and then every 6 months from years 3 to 5. Follow-up was mainly conducted by telephone and outpatient review. Physical examination, imaging studies, and blood tests (including tumor markers) were performed regularly in the outpatient clinic. DFS was calculated from the date of gastrectomy until the date of recurrence. OS was calculated from the date of diagnosis until the date of death or last follow-up. The last follow-up was performed on February 28, 2023.

### Statistical analysis

Categorical variables were presented as frequencies and percentages, and analyzed using either the Chi-square test or Fisher's exact test depending on the situation. Continuous variables were compared using the Mann–Whitney *U* test. Survival curves were plotted using the Kaplan–Meier method and compared using the log-rank test. A two-tailed *P-*value < 0.05 was considered statistically significant. All statistical analyses were conducted using SPSS (version 26.0; SPSS Inc., Chicago, IL, USA).

## Results

### Clinicopathological characteristics

In the entire cohort, 112 patients (74.7%) were men. The median age was 64 (range: 31–75) years. One hundred and thirty-nine patients (92.7%) had stage III disease. Most patients (96.0%) had gastric adenocarcinoma. Furthermore, 88.7% of tumors were poorly differentiated. Regarding tumor location, 83.3% of tumors were located in the stomach, while the remaining 16.7% were located at the gastroesophageal junction. Detailed information is provided in Table 1 and 2.

**Table 2:** Clinicopathological characteristics before and after propensity score matching (PSM)

Variable	Before PSM			After PSM		
	Group A (n = 79)	Group B (n = 160)	P-value	Group A (n = 75)	Group B (n = 75)	P-value
Radical resection			0.174*			1.000*
R0 resection	79 (100.0)	155 (96.9)		75 (100)	74 (98.7)	
Non-R0 resection	0 (0.0)	5 (3.1)		0 (0.0)	1 (1.3)	
Surgical procedures			0.910			1.000
Distal gastrectomy	31 (39.2)	64 (40.0)		29 (38.7)	29 (38.7)	
Total gastrectomy	48 (60.8)	96 (60.0)		46 (61.3)	46 (61.3)	
Reconstruction method			0.111*			0.153*
Billroth I	0 (0.0)	10 (6.3)		0 (0.0)	4 (5.3)	
Billroth II	31 (39.2)	54 (33.8)		29 (38.7)	25 (33.3)	
Roux-en-Y esophagojejunostomy	48 (60.8)	96 (60.0)		46 (61.3)	46 (61.3)	
Tumor location			0.703*			0.421
Upper	21 (26.6)	51 (31.9)		20 (26.7)	26 (34.7)	
Middle	22 (27.8)	39 (24.4)		22 (29.3)	16 (21.3)	
Lower	36 (45.6)	68 (42.5)		33 (44.0)	33 (44.0)	
Total stomach	0 (0.0)	2 (1.3)		0 (0.0)	0 (0.0)	
Pathologic stage (ypTNM)			< 0.001			0.006
ypT0N0M0	16 (20.3)	4 (2.5)		16 (21.3)	3 (4.0)	
I	19 (24.1)	34 (21.3)		18 (24.0)	14 (18.7)	
II	17 (21.5)	42 (26.3)		17 (22.7)	23 (30.7)	
III	27 (34.2)	80 (50.0)		24 (32.0)	35 (46.7)	
Differentiation degree			0.610			0.440
Poorly	70 (88.6)	138 (86.3)		68 (90.7)	65 (86.7)	
Moderately-highly	9 (11.4)	22 (13.8)		7 (9.3)	10 (13.3)	
Pathological type			0.290*			0.557*
Adenocarcinoma	74 (93.7)	156 (97.5)		71 (94.7)	73 (97.3)	
Signet-ring cell carcinoma	2 (2.5)	2 (1.3)		2 (2.7)	0 (0.0)	
Other^※^	3 (3.8)	2 (1.3)		2 (2.7)	2 (2.7)	
Nerve infiltration			0.722			0.479
No	51 (64.6)	107 (66.9)		50 (66.7)	54 (72.0)	
Yes	28 (35.4)	53 (33.1)		25 (33.3)	21 (28.0)	
Vascular tumor embolus			0.795			0.484
No	51 (64.6)	106 (66.3)		49 (65.3)	53 (70.7)	
Yes	28 (35.4)	54 (33.8)		26 (34.7)	22 (29.3)	
Postoperative CEA			0.309			0.290
≤ 5	67 (84.8)	143 (89.4)		65 (86.7)	69 (92.0)	
> 5	12 (15.2)	17 (10.6)		10 (13.3)	6 (8.0)	
Postoperative CA125			0.542			0.723
≤ 35	54 (68.4)	103 (64.4)		51 (68.0)	53 (70.7)	
> 35	25 (31.6)	57 (35.6)		24 (32.0)	22 (29.3)	
Postoperative CA199			0.688			0.575
≤ 37	71 (89.9)	141 (88.1)		67 (89.3)	69 (92.0)	
> 37	8 (10.1)	19 (11.9)		8 (10.7)	6 (8.0)	

*CEA* carcinoembryonic antigen, *CA125* carbohydrate antigen 125, *CA199* carbohydrate antigen199

*Fisher’s test

^※^Including adenosquamous carcinoma and squamous cell carcinoma

### Treatment outcomes

#### Perioperative treatment

In both groups, the median number of preoperative treatment cycles was three, and the median number of postoperative treatment cycles was four. After neoadjuvant treatment, all patients underwent D2 radical gastrectomy. R0 resection was performed in 75 patients (100%) in Group A and 74 patients (98.7%) in Group B, with no significant difference between the groups (*P* = 1.000, Table 2). Four patients in Group B underwent multivisceral resection due to tumor invasion or lymph node metastasis (splenectomy in three patients, pancreatectomy in two patients, and colectomy in one patient).

The median time between preoperative treatment and surgery was 29 (range 22–54) days in Group A and 30 (range: 21–49) days in Group B (*P* = 0.265). The median time between surgery and postoperative treatment was 27 (range 21–32) and 26 (range 21–29) days, respectively (*P* = 0.307).

In the entire cohort, surgical complications were observed in 16 patients (10.7%), including nine patients in Group A (pneumonia in five patients, intraperitoneal infection in two patients, and abdominal hemorrhage and anastomotic leakage in one patient each) and seven patients in Group B (pneumonia in three patients, anastomotic leakage in two patients, and intraperitoneal and wound infection in one patient each). There was no significant difference between the two groups (*P* = 0.597). The postoperative condition of these patients improved after symptomatic treatment. No surgery-related deaths occurred in either group.

#### Tumor regression and survival

Tumor responses were assessed in all patients. CR, PR, and SD were achieved in 5 (6.7%), 48 (64.0%), and 22 (29.3%) patients in group A, and 2 (2.7%), 46 (61.3%), and 27 (36.0%) patients in group B, respectively, without a significant difference (P = 0.434, Supplementary Table S1). After surgery, the pathological regression of resected specimens was evaluated according to the Becker criteria. In the entire cohort, 19 patients (12.7%) achieved pCR (TRG1a), including 16 patients (21.3%) in Group A and three patients (4.0%) in Group B, with a significant difference between the groups (*P* = 0.001). The MPR rate was significantly higher in Group A than in Group B (45.3% *vs*. 22.7%, respectively; *P* = 0.003). Significant downstaging of TNM stage and the T and N categories were observed in Group A. Detailed information is provided in Tables 3 and Supplementary Tables S2, S3.

**Table 3:** Pathological evaluation of response to neoadjuvant treatment in GC patients

Variable	Group A (No., %)	Group B (No.,%)	P-value
TRG			0.006
1a	16 (21.3)	3 (4.0)	
1b	18 (24.0)	14 (18.7)	
2	32 (42.7)	43 (57.3)	
3	9 (12.0)	15 (20.0)	
pCR	16 (21.3)	3 (4.0)	0.001
MPR	34 (45.3)	17 (22.7)	0.003

**Fig. 2:**
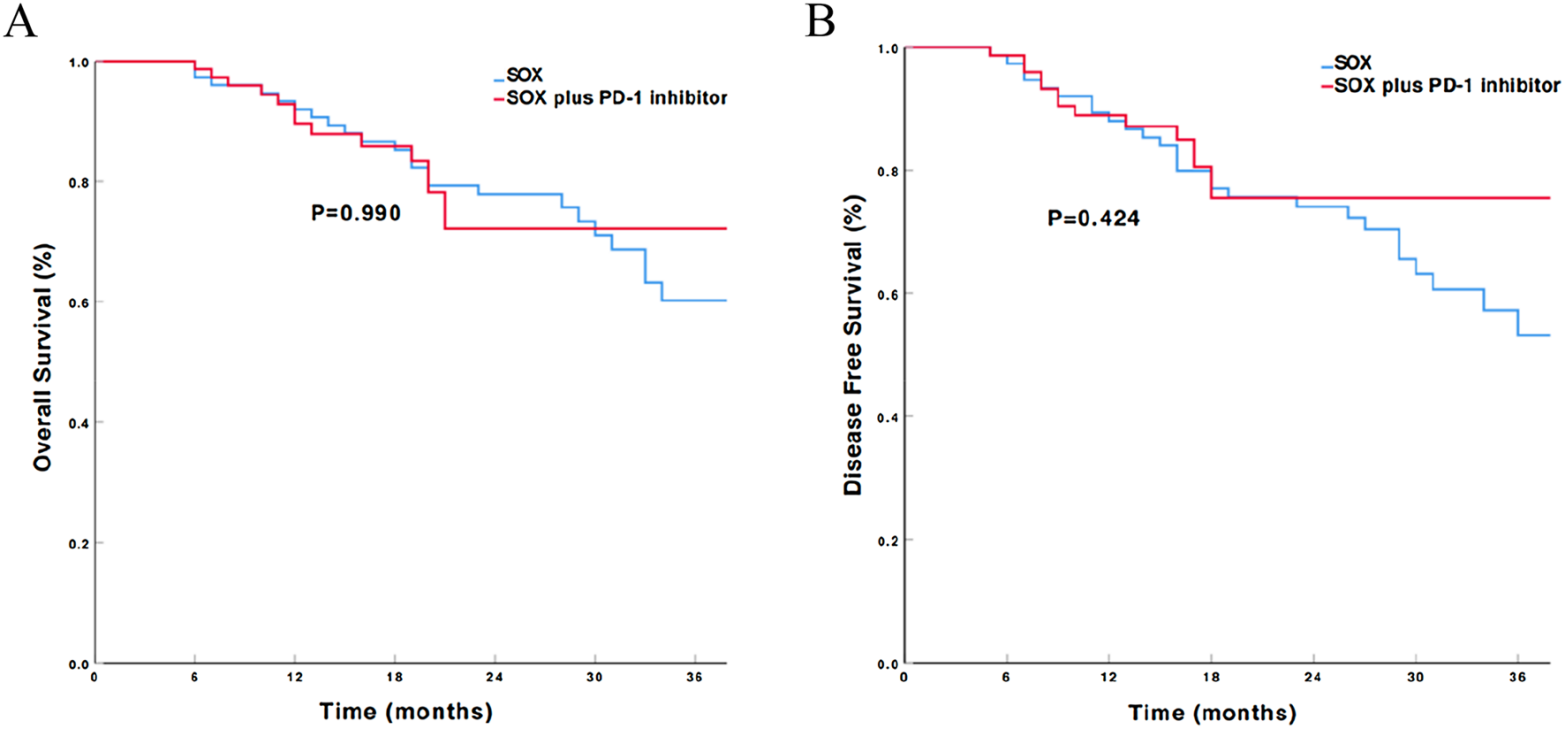
Kaplan–Meier estimates of overall survival (**A**) and disease free survival (**B**) of patients in group A and group B

All discharged patients were followed-up, with a median duration of 26 (range: 6–52) months. Median OS and DFS data are not yet mature. The 1-year OS rate was 92.8% for Group A and 92.0% for Group B, with no statistical difference between the groups (*P* = 0.392). The 1-year DFS rate for Group A and Group B was 88.9% and 88.0%, respectively (*P* = 0.357; Fig. 2).

### Safety profile

As shown in Table 4, grade 3 or 4 adverse events were identified in 17 (22.7%) patients in Group A and 13 (17.3%) patients in Group B during the neoadjuvant treatment period, with no significant difference (P = 0.414). Meanwhile, during the adjuvant treatment period, grade 3 or 4 adverse events were observed in 11 (14.7%) patients in Group A and 5 (6.7%) patients in Group B (P = 0.113). In the entire cohort, the most common hematological toxicities were leucopenia, neutropenia, and anemia, while the most common non-hematological toxicities were transaminase elevation, vomiting, and pneumonia. In Group A, two patients suffered from grade 3 hypothyroidism and were treated with thyroid hormone and steroid hormone. The condition of all patients with adverse events was well-controlled after conversion treatment. No treatment-related deaths occurred in either group.

**Table 4:** Perioperative treatment-related adverse events

	Neoadjuvant treatment				Adjuvant treatment			
	Group A (n.%)		Group B (n.%)		Group A (n.%)		Group B (n.%)	
	Any grade	Grade 3–4	Any grade	Grade 3–4	Any grade	Grade 3–4	Any grade	Grade 3–4
Total	70 (93.3)	17 (22.6)	67 (89.3)	13 (17.3)	68 (90.7)	11 (14.7)	64 (85.3)	5 (6.7)
Hematological								
Leucopenia	39 (52.0)	4 (5.3)	37 (49.3)	3 (4.0)	32 (42.7)	2 (2.7)	28 (37.3)	2 (2.7)
Neutropenia	33 (44.0)	4 (5.3)	34 (45.3)	3 (4.0)	36 (48.0)	3 (4.0)	23 (30.7)	1 (1.3)
Anemia	32 (42.7)	2 (2.7)	34 (45.3)	2 (2.7)	25 (33.3)	1 (1.3)	30 (40.0)	2 (2.7)
Thrombocytopenia	21 (28.0)	3 (4.0)	25 (33.3)	4 (5.3)	13 (17.3)	2 (2.7)	9 (12.0)	2 (2.7)
Non-hematological								
Elevated ALT/AST	18 (24.0)	4 (5.3)	19 (25.3)	3 (4.0)	21 (28.0)	3 (4.0)	13 (17.3)	1 (1.3)
Nausea/vomiting	15 (20.0)	2 (2.7)	13 (17.3)	1 (1.3)	8 (10.7)	1 (1.3)	10 (13.3)	1 (1.3)
Pneumonia	5 (6.7)	0 (0.0)	4 (5.3)	0 (0.0)	1 (1.3)	0 (0.0)	0 (0.0)	0 (0.0)
Diarrhea	5 (6.7)	0 (0.0)	3 (4.0)	0 (0.0)	1 (1.3)	0 (0.0)	2 (2.7)	0 (0.0)
Elevated creatinine	4 (5.3)	0 (0.0)	2 (2.7)	0 (0.0)	4 (5.3)	0 (0.0)	6 (8.0)	0 (0.0)
Dermatitis	2 (2.7)	0 (0.0)	0 (0.0)	0 (0.0)	1 (1.3)	0 (0.0)	0 (0.0)	0 (0.0)
Hypothyroidism	4 (5.3)	2 (2.7)	0 (0.0)	0 (0.0)	1 (1.3)	0 (0.0)	0 (0.0)	0 (0.0)
Hyperthyroidism	1 (1.3)	0 (0.0)	0 (0.0)	0 (0.0)	0 (0.0)	0 (0.0)	0 (0.0)	0 (0.0)

*ALT* alanine aminotransferase, *AST* aspartate aminotransferase

### Potential biomarkers

PD-L1 expression, MMR status, and EBV-positivity were analyzed in Group A patients. Detailed information is provided in Table 5 and Supplementary Tables S4–S6. PD-L1 data were available for 67 patients. The number of patients with a CPS < 1, 1 ≤ CPS < 5, 5 ≤ CPS < 10, and CPS ≥ 10 was 20 (29.9%), 15 (22.4%), 14 (20.9%), and 18 (26.9%), respectively. Forty-seven patients (70.1%) were PD-L1-positive (CPS ≥ 1). The pCR rate of PD-L1-positive and -negative patients was 27.7% and 15.0% (*P* = 0.215), and the MPR rate was 48.9% and 40.0% (*P* = 0.502), respectively. Further analysis showed that the pCR (40.6% *vs*. 8.6%; *P* = 0.002) and MPR (65.6% *vs*. 28.6%; *P* = 0.002) rates were higher in patients with a CPS ≥ 5 compared to those with a CPS < 5, with significant differences between the groups. MMR data were available for 71 patients, of whom seven (9.9%) had dMMR. Three patients (42.9%) with dMMR achieved pCR. EBV data were available for 65 patients. Only two patients (3.1%) were EBV-positive (one achieved pCR; the other achieved MPR). Both EBV-positive patients had a CPS of 5 and pMMR.

**Table 5:** Associations between the expression of PD-L1 and pathological response

Variable	CPS < 1 (n = 20)	CPS ≥ 1 (n = 47)	P-value	CPS < 5 (n = 35)	CPS ≥ 5 (n = 32)	P-value
TRG			0.738			0.008
1a	3 (15.0)	13 (27.7)		3 (8.6)	13 (40.6)	
1b	5 (25.0)	10 (21.3)		7 (20.0)	8 (25.0)	
2	9 (45.0)	18 (38.3)		19 (54.3)	8 (25.0)	
3	3 (15.0)	6 (12.8)		6 (17.1)	3 (9.4)	
pCR	3 (15.0)	13 (27.7)	0.215	3 (8.6)	13 (40.6)	0.002
MPR	8 (40.0)	23 (48.9)	0.502	10 (28.6)	21 (65.6)	0.002

## Discussion

In this study, we compared the efficacy of SOX plus a PD-1 inhibitor and SOX alone for the perioperative management of patients with stage IIb–III LAGC, focusing on pathological response and potential biomarkers. The pCR and downstaging rates of patients treated with perioperative SOX plus sintilimab were significantly higher than those of patients treated with SOX alone, which met the endpoints of this study as expected. Further analysis revealed that PD-L1 CPS ≥ 5, dMMR, or EBV-positivity were associated with improved efficacy.

The clinical benefit of neoadjuvant chemotherapy in patients with gastric cancer has been widely studied. Randomized trials reported that the addition of chemotherapy before gastrectomy improves the radical resection rate and prognosis compared to surgery alone [9, 10, 26, 27]. However, the pathological response rate of patients receiving neoadjuvant chemotherapy was relatively low, and the long-term survival was still unsatisfactory. Therefore, more effective strategies are needed to improve the efficacy and prognosis of LAGC.

Previous studies have demonstrated that immunotherapy may enhance the antitumor activity of chemotherapeutic drugs by exerting a synergistic effect [28, 29]. Furthermore, several studies have shown that the combination of chemotherapy and ICIs can improve the efficacy and prognosis of patients with advanced gastric cancer [11, 12, 30]. Encouraged by these results, studies focusing on the efficacy and safety of preoperative chemotherapy in combination with a PD-1 inhibitor were performed in patients with LAGC. Jiang et al. [16] evaluated the benefit of preoperative oxaliplatin/capecitabine plus sintilimab for patients with resectable gastric and gastroesophageal junction cancer in a single-arm phase II trial. Improved pCR (19.4%, 7/36) and MPR (47.2%, 17/36) rates were achieved compared to traditional neoadjuvant chemotherapy. In our study, the pCR and MPR rates of patients treated with perioperative SOX plus sintilimab were significantly higher than those of patients treated with SOX alone, showing superiority in the pathological response to this combination strategy directly based on the PD-1 inhibitor. However, for patients treated with neoadjuvant nivolumab monotherapy followed by gastrectomy, Hasegawa et al. [31] reported that the pCR (3.2%, 1/31) and MPR (16.1%, 5/31) rates were lower than those of patients treated with combination therapy, indicating that the effect of immunotherapy based on a single agent may be limited and the synergistic effect of immunotherapy and chemotherapy may yield better results. However, it remains unclear whether the superiority in pathological regression could translate into a survival benefit for patients with LAGC treated with chemotherapy and PD-1 inhibitors. Jiang et al. [16] showed that the 1-year OS and DFS rates in patients treated with preoperative chemotherapy plus sintilimab were 94.1% and 90.3%, respectively. In our study, the survival curves gradually separated during the follow-up period, however, there was no significant difference in the 1-year OS and DFS rates between the two groups. Long-term follow-up is needed to determine the survival benefit of perioperative chemotherapy plus immunotherapy in these patients. Moreover, the ongoing phase III KEYNOTE-585 trial of the perioperative XP/FP/FLOT regimen combined with pembrolizumab, with the primary endpoint of OS, will provide additional data in the future [32].

Regarding the safety of preoperative treatment, a meta-analysis reported that the incidence of grade 3–4 adverse events in patients treated with neoadjuvant chemoimmunotherapy and chemotherapy was 20.6% and 25.7%, respectively, with no significant difference (P = 0.380) [33]. Similarly, our results indicated that the addition of a PD-1 inhibitor to chemotherapy did not significantly increase the incidence of adverse events compared to chemotherapy alone. Therefore, the toxicity of PD-1 inhibitors in combination with chemotherapy may be tolerable. However, the potential immune-related adverse events (irAEs) should not be ignored. Previous studies indicate that organ-specific irAEs occur most frequently in the endocrine organs and skin, followed by the gastrointestinal and pulmonary tracts, most of which are grade 1–2 [16, 17]. In our study, a total of five patients (6.7%) in Group A experienced irAEs, including two cases of grade 3 hypothyroidism that required hormone replacement therapy. With the increased use of ICIs in the treatment of LAGC, it is important to be aware of potential adverse events and to become better at recognizing and managing these specific toxicities.

Biomarkers are increasingly driving systemic therapy. PD-L1 expression, MMR status, and EBV-positivity have been identified as potential biomarkers for immunotherapy benefit, however, evidence in the perioperative setting is still unclear [6]. Therefore, further exploratory analyses were performed in patients treated with PD-1 inhibitors in this study.

PD-L1 is a widely accepted biomarker for immunotherapy in gastric cancer. The CPS, which indicates PD-L1 expression levels on tumor cells and tumor-related immune cells, may be useful in predicting the efficacy of anti-PD-1 therapy [34]. However, the optimal cutoff value has not been established. Tang et al. [15] and Guo et al. [17] reported a higher pathological response rate in patients with a CPS ≥ 1 who were treated with preoperative chemoimmunotherapy. However, superior pCR and MPR rates were only observed in patients with a CPS ≥ 5 in the present study. Therefore, further research is needed to determine the appropriate cutoff value of PD-L1 CPS, which could improve patient selection for perioperative treatment.

Patients with microsatellite instability (MSI)/dMMR are characterized by increased PD-L1-positive T-cell infiltration and are more likely to respond to PD-1 inhibitors [35]. In this study, the pathological regression rate in patients with dMMR was higher than that in those with proficient MMR, indicating that the combination of chemotherapy and immunotherapy may be beneficial in these patients. However, whether patients with dMMR gastric cancer treated with perioperative chemoimmunotherapy have a superior prognosis compared to patients treated with chemotherapy alone is not clear, and there is still a lack of consensus on the treatment of these patients. An exploratory analysis of the MAGIC trial [36] revealed that preoperative chemotherapy may exert a negative effect on the prognosis of patients with MSI/dMMR LAGC. Meanwhile, the GERCOR NEONIPIGA phase II trial [37] reported a promising pCR rate (58.6%, 17/29) in patients with resectable MSI/dMMR gastric/gastroesophageal junction adenocarcinoma (T2–4NxM0) who were treated with neoadjuvant nivolumab plus ipilimumab. However, the most effective perioperative strategies for the management of patients with MSI/dMMR gastric cancer remain controversial, and further clinical studies are warranted.

As a distinct molecular subtype of gastric cancer, EBV-positivity has also been reported as a biomarker for anti-PD-1 therapy. Previous studies have indicated that EBV-associated gastric cancer (EBVaGC) exhibits higher expression of PD-L1 and lymphocytic infiltration [38, 39]. Patients with EBVaGC may be more susceptible to PD-1 blockade and may have a better prognosis [40–42]. Tang et al. [15] reported an exceptional pCR rate of 33.3% (2/6) in patients with EBVaGC who were treated with neoadjuvant PD-1 blockade plus chemotherapy. In this study, both patients with EBV-positive gastric cancer had encouraging outcomes: one achieved MPR and the other achieved pCR. Therefore, patients with EBVaGC may be an additional population with high potential for clinical benefit to chemoimmunotherapy.

Although the baseline characteristics were balanced and comparable between the two groups after PSM, there are several limitations in the present study. First, this was a single-center study, and selection bias was unavoidable given the retrospective design of the study. Second, the safety results of perioperative chemotherapy plus PD-1 inhibitor may be compromised due to the exclusion of some patients with incomplete treatment. Third, long-term survival and recurrence could not be fully analyzed due to the relatively short follow-up period. Finally, we did not explore the efficacy of perioperative chemotherapy plus PD-1 inhibitors in patients with different genomic backgrounds (tumor mutation burden, high microsatellite instability, etc.). Despite these limitations, our results are valuable for future research on the perioperative management of patients with LAGC.

In conclusion, perioperative SOX plus sintilimab may improve tumor downstaging and pathological regression rate of LAGC. Patients with PD-L1 CPS ≥ 5, EBV-positivity, or dMMR responded better to chemoimmunotherapy. However, long-term survival requires further follow-up, and more prospective clinical studies with larger sample sizes are needed to validate the findings of our study.

### Supplementary Information

Below is the link to the electronic supplementary material.

Supplementary file1 (DOCX 25 KB)

## Abbreviations

BSA: Body surface area
CPS: Combined positive score
DFS: Disease-free survival
dMMR: MMR deficiency
EBV: Epstein–Barr virus
EBVaGC: EBV-associated gastric cancer
ICI: Immune checkpoint inhibitor
irAEs: Immune-related adverse events
LAGC: Locally advanced gastric cancer
MLH1: MutL homolog 1
MMR: Mismatch repair
MPR: Major pathological response
MSH2: MutS homolog 2
MSH6: MutS homolog 6
MSI: Microsatellite instability
OS: Overall survival
pCR: Pathological complete response
PD-1: Programmed cell death protein-1
PD-L1/2: Programmed death ligand-1/2
PMS2: PMS1 homolog 2
PSM: Propensity score matching
TRG: Tumor regression grading

## References

[1] Sung H, Ferlay J, Siegel RL, Laversanne M, Soerjomataram I, Jemal A (2021). Global Cancer Statistics 2020: GLOBOCAN Estimates of Incidence and Mortality Worldwide for 36 Cancers in 185 Countries. CA Cancer J Clin.

[2] Smyth EC, Nilsson M, Grabsch HI, van Grieken NC, Lordick F (2020). Gastric cancer. Lancet.

[3] Ajani JA, D'Amico TA, Bentrem DJ, Chao J, Cooke D, Corvera C (2022). Gastric Cancer, Version 2.2022, NCCN Clinical Practice Guidelines in Oncology. J Natl Compr Canc Netw..

[4] Thomassen I, van Gestel YR, van Ramshorst B, Luyer MD, Bosscha K, Nienhuijs SW (2014). Peritoneal carcinomatosis of gastric origin: a population-based study on incidence, survival and risk factors. Int J Cancer.

[5] Chen QY, Zhong Q, Zhou JF, Qiu XT, Dang XY, Cai LS (2020). Conditional survival and recurrence of remnant gastric cancer after surgical resection: a multi-institutional study. Cancer Sci.

[6] Joshi SS, Badgwell BD (2021). Current treatment and recent progress in gastric cancer. CA Cancer J Clin.

[7] Al-Batran SE, Homann N, Pauligk C, Goetze TO, Meiler J, Kasper S (2019). Perioperative chemotherapy with fluorouracil plus leucovorin, oxaliplatin, and docetaxel versus fluorouracil or capecitabine plus cisplatin and epirubicin for locally advanced, resectable gastric or gastro-oesophageal junction adenocarcinoma (FLOT4): a randomised, phase 2/3 trial. Lancet.

[8] Zhang X, Liang H, Li Z, Xue Y, Wang Y, Zhou Z (2021). Perioperative or postoperative adjuvant oxaliplatin with S-1 versus adjuvant oxaliplatin with capecitabine in patients with locally advanced gastric or gastro-oesophageal junction adenocarcinoma undergoing D2 gastrectomy (RESOLVE): an open-label, superiority and non-inferiority, phase 3 randomised controlled trial. Lancet Oncol.

[9] Cunningham D, Allum WH, Stenning SP, Thompson JN, Van de Velde CJ, Nicolson M (2006). Perioperative chemotherapy versus surgery alone for resectable gastroesophageal cancer. N Engl J Med.

[10] Kang YK, Yook JH, Park YK, Lee JS, Kim YW, Kim JY (2021). PRODIGY: a phase III study of neoadjuvant docetaxel, oxaliplatin, and S-1 plus surgery and adjuvant S-1 versus surgery and adjuvant S-1 for resectable advanced gastric cancer. J Clin Oncol.

[11] Kang YK, Chen LT, Ryu MH, Oh DY, Oh SC, Chung HC (2022). Nivolumab plus chemotherapy versus placebo plus chemotherapy in patients with HER2-negative, untreated, unresectable advanced or recurrent gastric or gastro-oesophageal junction cancer (ATTRACTION-4): a randomised, multicentre, double-blind, placebo-controlled, phase 3 trial. Lancet Oncol.

[12] Janjigian YY, Shitara K, Moehler M, Garrido M, Salman P, Shen L (2021). First-line nivolumab plus chemotherapy versus chemotherapy alone for advanced gastric, gastro-oesophageal junction, and oesophageal adenocarcinoma (CheckMate 649): a randomised, open-label, phase 3 trial. Lancet.

[13] Boku N, Ryu MH, Kato K, Chung HC, Minashi K, Lee KW (2019). Safety and efficacy of nivolumab in combination with S-1/capecitabine plus oxaliplatin in patients with previously untreated, unresectable, advanced, or recurrent gastric/gastroesophageal junction cancer: interim results of a randomized, phase II trial (ATTRACTION-4). Ann Oncol.

[14] Bagchi S, Yuan R, Engleman EG (2021). Immune checkpoint inhibitors for the treatment of cancer: clinical impact and mechanisms of response and resistance. Annu Rev Pathol.

[15] Tang X, Li M, Wu X, Guo T, Zhang L, Tang L (2022). Neoadjuvant PD-1 blockade plus chemotherapy induces a high pathological complete response rate and anti-tumor immune subsets in clinical stage III gastric cancer. Oncoimmunology.

[16] Jiang H, Yu X, Li N, Kong M, Ma Z, Zhou D (2022). Efficacy and safety of neoadjuvant sintilimab, oxaliplatin and capecitabine in patients with locally advanced, resectable gastric or gastroesophageal junction adenocarcinoma: early results of a phase 2 study. J Immunother Cancer..

[17] Guo H, Ding P, Sun C, Yang P, Tian Y, Liu Y (2022). Efficacy and safety of sintilimab plus XELOX as a neoadjuvant regimen in patients with locally advanced gastric cancer: a single-arm, open-label, phase II trial. Front Oncol.

[18] Xu C, Xie X, Kang N, Jiang H (2022). Neoadjuvant PD-1 inhibitor and apatinib combined with S-1 plus oxaliplatin for locally advanced gastric cancer patients: a multicentered, prospective, cohort study. J Cancer Res Clin Oncol..

[19] Japanese Gastric Cancer Association (2017). Japanese gastric cancer treatment guidelines 2014 (ver. 4). Gastric Cancer.

[20] Japanese gastric cancer treatment guidelines 2018 (5th edition). Gastric Cancer. 2021;24:1–21. 10.1007/s10120-020-01042-y PMC7790804 32060757

[21] Hermanek P, Wittekind C (1994). Residual tumor (R) classification and prognosis. Semin Surg Oncol.

[22] Dindo D, Demartines N, Clavien PA (2004). Classification of surgical complications: a new proposal with evaluation in a cohort of 6336 patients and results of a survey. Ann Surg.

[23] Common terminology criteria for adverse events version 3.0 (CTCAE V.3.0). http://ctep.cancer.gov/protocolDevelopment/electronic_applications/ctc.htm.

[24] Eisenhauer EA, Therasse P, Bogaerts J, Schwartz LH, Sargent D, Ford R (2009). New response evaluation criteria in solid tumours: revised RECIST guideline (version 1.1). Eur J Cancer..

[25] Becker K, Mueller JD, Schulmacher C, Ott K, Fink U, Busch R (2003). Histomorphology and grading of regression in gastric carcinoma treated with neoadjuvant chemotherapy. Cancer.

[26] Ychou M, Boige V, Pignon JP, Conroy T, Bouché O, Lebreton G (2011). Perioperative chemotherapy compared with surgery alone for resectable gastroesophageal adenocarcinoma: an FNCLCC and FFCD multicenter phase III trial. J Clin Oncol.

[27] Schuhmacher C, Gretschel S, Lordick F, Reichardt P, Hohenberger W, Eisenberger CF (2010). Neoadjuvant chemotherapy compared with surgery alone for locally advanced cancer of the stomach and cardia: European Organisation for Research and Treatment of Cancer randomized trial 40954. J Clin Oncol.

[28] Principe DR, Kamath SD, Korc M, Munshi HG (2022). The immune modifying effects of chemotherapy and advances in chemo-immunotherapy. Pharmacol Ther.

[29] Kim R, An M, Lee H, Mehta A, Heo YJ, Kim KM (2022). Early tumor-immune microenvironmental remodeling and response to first-line fluoropyrimidine and platinum chemotherapy in advanced gastric cancer. Cancer Discov.

[30] Janjigian YY, Kawazoe A, Yañez P, Li N, Lonardi S, Kolesnik O (2021). The KEYNOTE-811 trial of dual PD-1 and HER2 blockade in HER2-positive gastric cancer. Nature.

[31] Hasegawa H, Shitara K, Takiguchi S, Takiguchi N, Ito S, Kochi M (2022). A multicenter, open-label, single-arm phase I trial of neoadjuvant nivolumab monotherapy for resectable gastric cancer. Gastric Cancer.

[32] Bang YJ, Van Cutsem E, Fuchs CS, Ohtsu A, Tabernero J, Ilson DH (2019). KEYNOTE-585: Phase III study of perioperative chemotherapy with or without pembrolizumab for gastric cancer. Future Oncol.

[33] Xu H, Li T, Shao G, Wang W, He Z, Xu J (2023). Evaluation of neoadjuvant immunotherapy plus chemotherapy in Chinese surgically resectable gastric cancer: a pilot study by meta-analysis. Front Immunol.

[34] Shitara K, Özgüroğlu M, Bang YJ, Di Bartolomeo M, Mandalà M, Ryu MH (2018). Pembrolizumab versus paclitaxel for previously treated, advanced gastric or gastro-oesophageal junction cancer (KEYNOTE-061): a randomised, open-label, controlled, phase 3 trial. Lancet.

[35] Le DT, Uram JN, Wang H, Bartlett BR, Kemberling H, Eyring AD (2015). PD-1 blockade in tumors with mismatch-repair deficiency. N Engl J Med.

[36] Smyth EC, Wotherspoon A, Peckitt C, Gonzalez D, Hulkki-Wilson S, Eltahir Z (2017). Mismatch repair deficiency, microsatellite instability, and survival: an exploratory analysis of the medical research council adjuvant gastric infusional chemotherapy (MAGIC) trial. JAMA Oncol.

[37] André T, Tougeron D, Piessen G, Fouchardière CDL, Louvet C, Adenis A (2023). Neoadjuvant nivolumab plus ipilimumab and adjuvant nivolumab in localized deficient mismatch repair/microsatellite instability-high gastric or esophagogastric junction adenocarcinoma: the GERCOR NEONIPIGA phase II study. J Clin Oncol..

[38] Lima Á, Sousa H, Medeiros R, Nobre A, Machado M (2022). PD-L1 expression in EBV associated gastric cancer: a systematic review and meta-analysis. Discov Oncol.

[39] Panda A, Mehnert JM, Hirshfield KM, Riedlinger G, Damare S, Saunders T (2018). Immune activation and benefit from avelumab in EBV-positive gastric cancer. J Natl Cancer Inst.

[40] Cancer Genome Atlas Research Network (2014). Comprehensive molecular characterization of gastric adenocarcinoma. Nature.

[41] Greally M, Chou JF, Chatila WK, Margolis M, Capanu M, Hechtman JF (2019). Clinical and molecular predictors of response to immune checkpoint inhibitors in patients with advanced esophagogastric cancer. Clin Cancer Res.

[42] Sohn BH, Hwang JE, Jang HJ, Lee HS, Oh SC, Shim JJ (2017). Clinical significance of four molecular subtypes of gastric cancer identified by the cancer genome atlas project. Clin Cancer Res.

