# Supplementary material for: SOX combined with sintilimab versus SOX alone in the perioperative management of locally advanced gastric cancer: a propensity score–matched analysis
Source: Gastric Cancer. 2023 Sep 28;26(6):1040–50. doi: 10.1007/s10120-023-01431-z (PMC10640399; doi:10.1007/s10120-023-01431-z)
Supplement: Supplementary file 1 — Supplementary file1 (DOCX 25 KB) [file 10120_2023_1431_MOESM1_ESM.docx]

**SOX combined with sintilimab versus SOX alone in the perioperative management of locally advanced gastric cancer: a propensity score–matched analysis**

**Xingmao Huang^1,2^*, Jingquan Fang^3,1^*, Ling Huang^1^*, Hang Chen^1,2^, Han Chen^1,2^, Tengjiao Chai^1^, Zeyao Ye^1^, Hanguang Chen^4^, Qi Xu^5^, Yian Du^1^, Pengfei Yu^1^#**

1 Department of Gastric Surgery, Zhejiang Cancer Hospital, Hangzhou Institute of Medicine (HIM), Chinese Academy of Sciences, Hangzhou, Zhejiang 310022, China.

2 Postgraduate training base Alliance of Wenzhou Medical University (Zhejiang Cancer Hospital), Hangzhou, Zhejiang 310022, China.

3 Zhejiang Chinese Medical University, Hangzhou, Zhejiang 310053, China.

4 Department of General surgery, No.2 People’s Hospital of Yuhang District, Hangzhou, Zhejiang 310022, China.

5 Department of Medical Oncology, Zhejiang Cancer Hospital, Hangzhou Institute of Medicine (HIM), Chinese Academy of Sciences, Hangzhou, Zhejiang 310022, China.

* These authors contributed equally to this work and should be considered as co-first authors.

# Corresponding author: Pengfei Yu, M.D. Phone: +8613588369423 Email: ypfzmu@163.com. Department of Gastric Surgery, Zhejiang Cancer Hospital, Hangzhou Institute of Medicine (HIM), Chinese Academy of Sciences, Hangzhou, Zhejiang 310022, China.

Article type: original article

Running head: SOX plus sintilimab in LAGC patients

Submitted to Journal: Gastric Cancer

Supplementary Table S1. Clinical response of neoadjuvant treatment in GC patients in group A and group B

| **Variable** | **Group A (No.,%)** | **Group B (No.,%)** | **P-Value** |
| --- | --- | --- | --- |
| **CR** | 5(6.7) | 2(2.7) | 0.434 |
| **PR** | 48(64.0) | 46(61.3) |  |
| **SD** | 22(29.3) | 27(36.0) |  |

Supplementary Table S2. Tumor downstaging rate of GC patients

| **Clinical stage before treatment** | **Pathological stage after operation** | **Group A (No.，%)** | **Group B**  **(No.,%)** | **P-Value** |
| --- | --- | --- | --- | --- |
| **T-stage** |  |  |  | 0.031 |
| cT3 | ypT0 | 5 (6.7) | 1 (1.3) |  |
|  | ypT1 | 6 (8.0) | 4 (5.3) |  |
|  | ypT2 | 3 (4.0) | 4 (5.3) |  |
|  | ypT3 | 6 (8.0) | 12 (16.0) |  |
|  | ypT4 | 2 (2.7) | 4 (5.3) |  |
| cT4 | ypT0 | 11 (14.7) | 2 (2.7) |  |
|  | ypT1 | 7 (9.3) | 5 (6.7) |  |
|  | ypT2 | 8 (10.7) | 14 (18.7) |  |
|  | ypT3 | 11 (14.7) | 8 (10.7) |  |
|  | ypT4 | 16 (21.3) | 21 (28.0) |  |
| **N-stage** |  |  |  | 0.010 |
| cN0 | ypN0 | 4 (5.3) | 4 (5.3) |  |
|  | ypN1 | 1 (1.3) | 0 (0.0) |  |
|  | ypN2 | 0 (0.0) | 1 (1.3) |  |
|  | ypN3 | 0 (0.0) | 1 (1.3) |  |
| cN1 | ypN0 | 3 (4.0) | 4 (5.3) |  |
|  | ypN1 | 2 (2.7) | 1 (1.3) |  |
|  | ypN2 | 1 (1.3) | 1 (1.3) |  |
|  | ypN3 | 1 (1.3) | 0 (0.0) |  |
| cN2 | ypN0 | 16 (21.3) | 17 (22.7) |  |
|  | ypN1 | 8 (10.7) | 14 (18.7) |  |
|  | ypN2 | 2 (2.7) | 9 (12.0) |  |
|  | ypN3 | 2 (2.7) | 4 (5.3) |  |
| cN3 | ypN0 | 17 (22.7) | 3 (4.0) |  |
|  | ypN1 | 6 (8.0) | 2 (2.7) |  |
|  | ypN2 | 6 (8.0) | 1 (1.3) |  |
|  | ypN3 | 6 (8.0) | 13 (17.3) |  |
| **TNM-stage** |  |  |  | 0.047 |
| cIIb | ypT0N0M0 | 3 (4.0) | 1 (1.3) |  |
|  | yp stage I | 1 (1.3) | 2 (2.7) |  |
|  | yp stage II | 1 (1.3) | 2 (2.7) |  |
|  | yp stage III | 0 (0.0) | 1 (1.3) |  |
| cIII | ypT0N0M0 | 13 (17.3) | 2 (2.7) |  |
|  | yp stage I | 17 (22.7) | 12 (16.0) |  |
|  | yp stage II | 16 (21.3) | 21 (28.0) |  |
|  | yp stage III | 24 (32.0) | 34 (45.3) |  |

Supplementary Table S3. Pathological responses of patients with different treatment cycles in Group A and Group B

| **Variable** | **Number of cycles** | **TRG1a** | **TRG1b** | **TRG2** | **TRG3** | **P-Value** |
| --- | --- | --- | --- | --- | --- | --- |
| **Group A** | 2 | 3(4.0) | 6(8.0) | 7(9.3) | 2(2.7) | 0.977 |
|  | 3 | 9(12.0) | 8(10.7) | 18(24.0) | 5(6.7) |  |
|  | 4 | 4(5.3) | 4(5.3) | 7(9.3) | 2(2.7) |  |
| **Group B** | 2 | 0(0.0) | 6(8.0) | 21(28.0) | 3(4.0) | 0.071 |
|  | 3 | 1(1.3) | 6(8.0) | 18(24.0) | 7(9.3) |  |
|  | 4 | 2(2.7) | 2(2.7) | 4(5.3) | 5(6.7) |  |

Supplementary Table S4. Associations between the expression of PD-L1 and pathological response

| **Variable** | **CPS<1**  **(n=20)** | **CPS [1,5)**  **(n=15)** | **CPS [5,10)**  **(n=14)** | **CPS≥10**  **(n=18)** | **P-Value** |
| --- | --- | --- | --- | --- | --- |
| **TRG** |  |  |  |  | 0.030 |
| 1a | 3(15.0) | 0(0.0) | 5(35.7) | 8(44.4) |  |
| 1b | 5(25.0) | 2(13.3) | 5(35.7) | 3(16.7) |  |
| 2 | 9(45.0) | 10(66.7) | 2(14.3) | 6(33.3) |  |
| 3 | 3(15.0) | 3(20.0) | 2(14.3) | 1(5.6) |  |
| **pCR** | 3(15.0) | 0(0.0) | 5(35.7) | 8(44.4) | 0.008 |
| **MPR** | 8(40.0) | 2(13.3) | 10(71.4) | 11(61.1) | 0.007 |

Supplementary Table S5. Associations between the MMR status and pathological response

| **Variable** | **dMMR**  **(n=7)** | **pMMR**  **(n=64)** | **P-Value** |
| --- | --- | --- | --- |
| **TRG** |  |  | 0.354 |
| 1a | 3(42.9) | 13(20.3) |  |
| 1b | 0(0.0) | 16(25.0) |  |
| 2 | 3(42.9) | 27(42.2) |  |
| 3 | 1(14.3) | 8(12.5) |  |
| **pCR** | 3(42.9) | 13(20.3) | 0.185 |
| **MPR** | 3(42.9) | 29(45.3) | 0.612 |

Supplementary Table S6. Associations between the EBV status and pathological response

| **Variable** | **EBV negative**  **(n=63)** | **EBV positive**  **(n=2)** | **P-Value** |
| --- | --- | --- | --- |
| **TRG** |  |  | 0.270 |
| 1a | 10(15.9) | 1(50.0) |  |
| 1b | 13(20.6) | 1(50.0) |  |
| 2 | 31(49.2) | 0(0.0) |  |
| 3 | 9(14.3) | 0(0.0) |  |
| **pCR** | 10(15.9) | 1(50.0) | 0.312 |
| **MPR** | 23(36.5) | 2(100.0) | 0.144 |
